# Supplementary material for: Finely manipulating room temperature phosphorescence by dynamic lanthanide coordination toward multi-level information security
Source: Nat Commun. 2024 May 8;15:3846. doi: 10.1038/s41467-024-47674-x (PMC11078970; doi:10.1038/s41467-024-47674-x)
Supplement: Supplementary file 1 — Supplementary Information [file 41467_2024_47674_MOESM1_ESM.pdf]

Supplementary Information

Finely Manipulating Room Temperature Phosphorescence by Dynamic Lanthanide Coordination toward Multi-Level Information Security

Longqiang Li, Jiayin Zhou, Junyi Han, Depeng Liu, Min Qi, Juanfang Xu, Guangqiang Yin, Tao Chen**

L. Li, J. Zhou, J. Han, D. Liu, M. Qi, J. Xu, G. Yin, T. Chen
Key Laboratory of Marine Materials and Related Technologies, Zhejiang Key Laboratory of Marine Materials and Protective Technologies, Ningbo Institute of Materials Technology and Engineering, Chinese Academy of Sciences, Ningbo 315201, China.
E-mail: yinguangqiang@nimte.ac.cn; tao.chen@nimte.ac.cn

L. Li, J. Zhou, J. Han, D. Liu, M. Qi, J. Xu, G. Yin, T. Chen
School of Chemical Sciences, University of Chinese Academy of Sciences, Beijing 100049, China.

T. Chen
College of Material Chemistry and Chemical Engineering, Key Laboratory of Organosilicon Chemistry and Material Technology, Ministry of Education, Hangzhou Normal University, Hangzhou, 311121, Zhejiang, China.

1. Supplementary Methods.

Measurements The spectra of ^{1}H nuclear magnetic resonance (NMR) were obtained by a Bruker AVANCE 400 MHz spectrometer. X-ray photoelectron spectroscopy (XPS) was carried out with AXIS SUPRA. Powder X-ray diffraction (PXRD) was performed on a Bruker D8 ADVANCE DAVINCI. Fourier transforms infrared (FTIR) spectra were measured by a Thermo NICOLET-6700 FTIR spectrometer. UV-Vis absorption and transmittance spectra were recorded on a UV-Vis spectrophotometer (TU-1810, Purkunjie General Instrument Co., Ltd). At room temperature, the fluorescence spectra, phosphorescence spectra and phosphorescence-decay profiles were obtained by a Hitachi F-4600 fluorescence spectrofluorometer with a 150 W Xenon lamp. The excitation wavelength was 300 nm with a scan speed of 1200 nm/min. The final lifetimes(τ) were obtained by fitting the decay curve with a multiexponential decay function:

$$\mathrm{I}(t) = \sum_i A_i \, \mathrm{e}^{-\frac{t}{\tau_i}} \qquad (1)$$

where A_i and τ_i represent the amplitude and lifetime of an individual component for multiexponential decay profiles, respectively.

The average lifetimes were calculated according to the following equation:

$$\tau_{\mathrm{avg}} = \frac{\sum \alpha_i \tau_i^2}{\alpha_i \tau_i} \qquad (2)$$

Theoretical Calculations. The ground and excited-state optimizations were calculated using the Density Functional Theory (DFT) and time-dependent Density Functional Theory (TD-DFT) at the PBE0-D3[1]/6-311G(d,p)[2] + SDD[3] level with Gaussian 16, respectively. Here, the SDD effective core potential was used to describe the atomic orbital and relativistic effect of the heavy element Europium.

Synthesis of Ph-TPY. Successively added NaOH powder (2.1 g, 54 mmol), benzaldehyde (1 g, 9.7 mmol), and 2-acetylpyridine (2.6 g, 21.4 mmol) to 50 mL of ethanol. After stirring at room temperature for 10 h, add 35 mL aqueous $NH_3 \cdot H_2O$ to the mixture and reflux for 10 h. Then, the precipitate was fully washed with ethanol, and dried to obtain a light yellow powder (1.8 g, 60%). ^{1}H NMR (400 MHz, $CDCl_3$) δ 8.78 (s, 2H), 8.76 (d, J = 5.4 Hz, 2H), 8.71 (d, J = 8.0 Hz, 2H), 7.92 (ddd, J = 9.4, 8.3, 1.5 Hz, 4H), 7.54 (t, J = 7.3 Hz, 2H), 7.48 (t, J = 7.3 Hz, 1H), 7.38 (dd, J = 7.4, 4.9 Hz, 2H).

4TPYBOH@PVA-Ln film with white light emission: The 8.0 mL homogeneous precursor solution of 0.6 wt% 4TPYBOH@PVA was heated at 100 °C for 12 hours. After all solvents have completely evaporated, 8 mL H_2O, the mixture of $Eu(NO_3)_3 \cdot 6H_2O$ and $Tb(NO_3)_3 \cdot 5H_2O$ (the molar ratio of Eu^{3+} and Tb^{3+} is 1:3, the total mass is 1.5 mg, 3.0 mg, 4.0 mg, 5.0 mg, 6.0 mg) were added into the dry 4TPYBOH@PVA and heated at 96 °C for 3 hours until thoroughly dissolved. Then, 2.0 mL of the above Ln^{3+}-doped precursor solution was taken and drop-casted on a glass slide (25 mm×75 mm). Finally, the glass slide was then heated at 65 °C for 2 hours to obtain Ln^{3+}-doped RTP film with white light emission.

4TPYBOH@PAN-Eu films: The mixed solution of 500 mg of polyacrylonitrile (PAN), 3.0 mg of 4TPYBOH powder and $Eu(NO_3)_3 \cdot 6H_2O$ (0 mg, 1.0 mg, 2.0 mg, 5.0 mg) in 8.0 mL of N, N-Dimethylformamide (DMF) was heated at 45 °C under stirring for 90 minutes to obtain the homogeneous precursor solution. Then, 2.0 mL of the homogeneous precursor solution was taken and drop-casted on a glass slide (25 mm×75 mm). Finally, 4TPYBOH@PAN-Eu films were obtained by heating the resulting glass slide at 50 °C for 4 hours to remove DMF completely.

The solution of $(4TPYBOH)_2$-Eu^{3+}-COT: 50.0 mg of 4TPYBOH (0.14 mmol) and 31.6 mg of $Eu(NO_3)_3 \cdot 6H_2O$ (0.07 mmol) were dissolved in 20.0 mL of DMF to obtain the homogeneous $(4TPYBOH)_2$-Eu^{3+} solution. After stirring at room temperature for 1 minute, 2.0 mL of $(4TPYBOH)_2$-Eu^{3+} solution was taken and mixed with different additions of COT liquid (0.5, 1.0, 2.0, 3.0, 4.0, 5.0 μL) to obtain $(4TPYBOH)_2$-Eu^{3+}-COT. The fluorescence spectra were measured for $(4TPYBOH)_2$-Eu^{3+} solution and $(4TPYBOH)_2$-Eu^{3+}-COT under 254 nm excitation.

2. Supplementary Figures

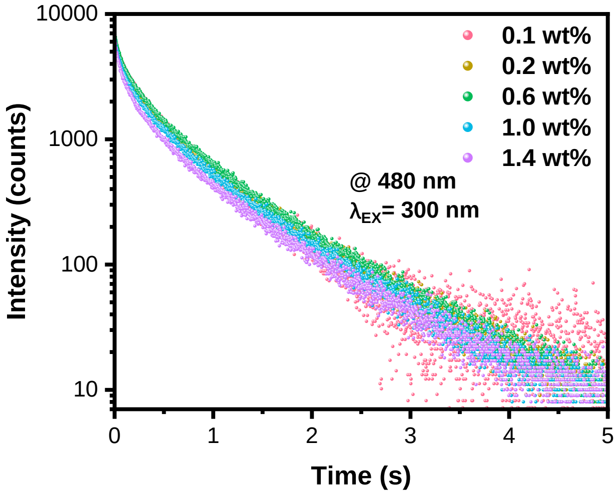

Supplementary Figure 1. Phosphorescence-decay profiles of 4TPYBOH@PVA films at different doping ratios of organic phosphor at 480 nm.

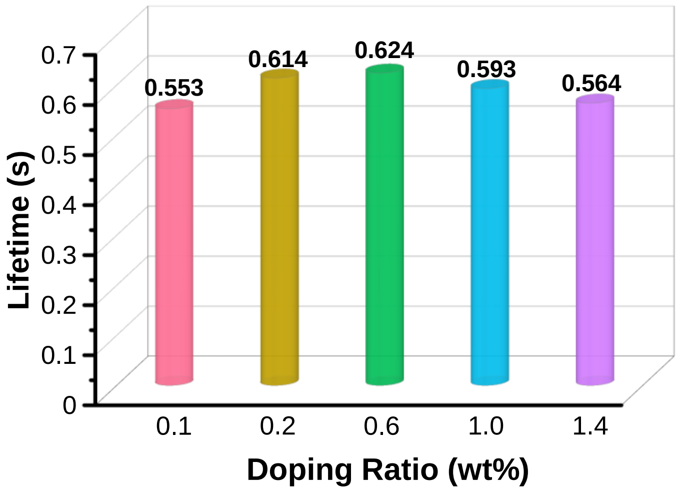

Supplementary Figure 2. Lifetime histogram (monitored at 480 nm) of 4TPYBOH@PVA films at different doping ratios of organic phosphor.

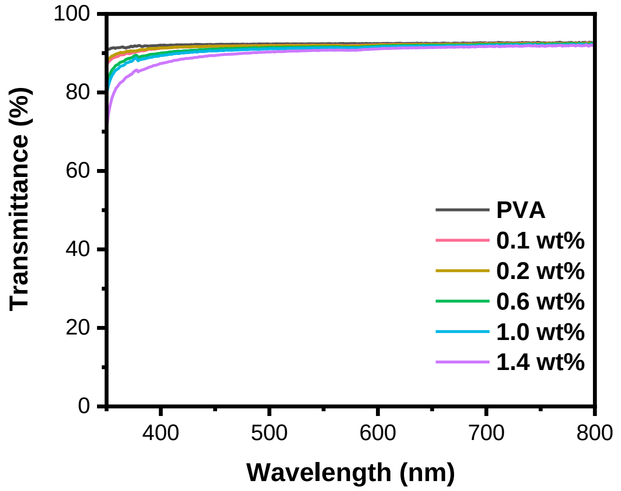

Supplementary Figure 3. Transmittance spectra of 4TPYBOH@PVA films at different doping ratios of organic phosphor.

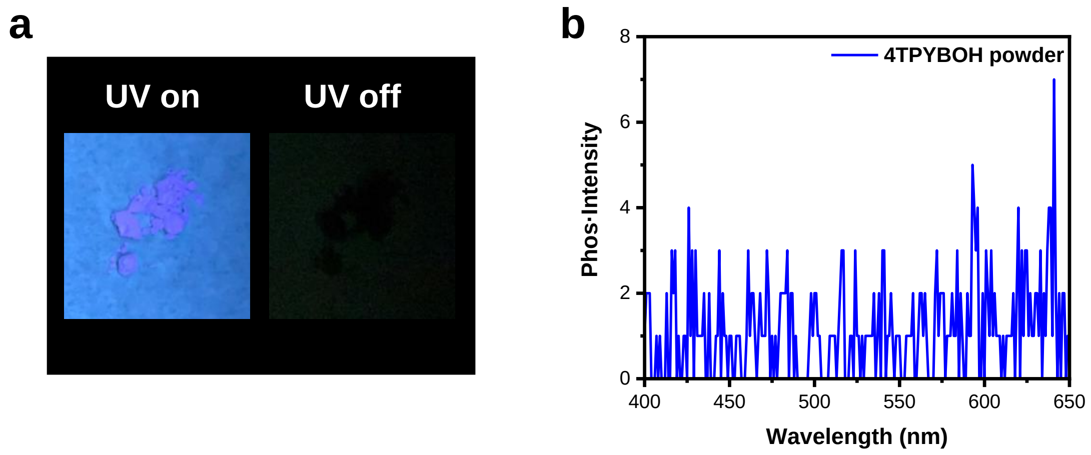

Supplementary Figure 4. (a) Photographs of 4TPYBOH powder taken under and after removing the 254 nm UV irradiation. (b) Phosphorescence spectra of 4TPYBOH powder with a delay time of 100 ms.

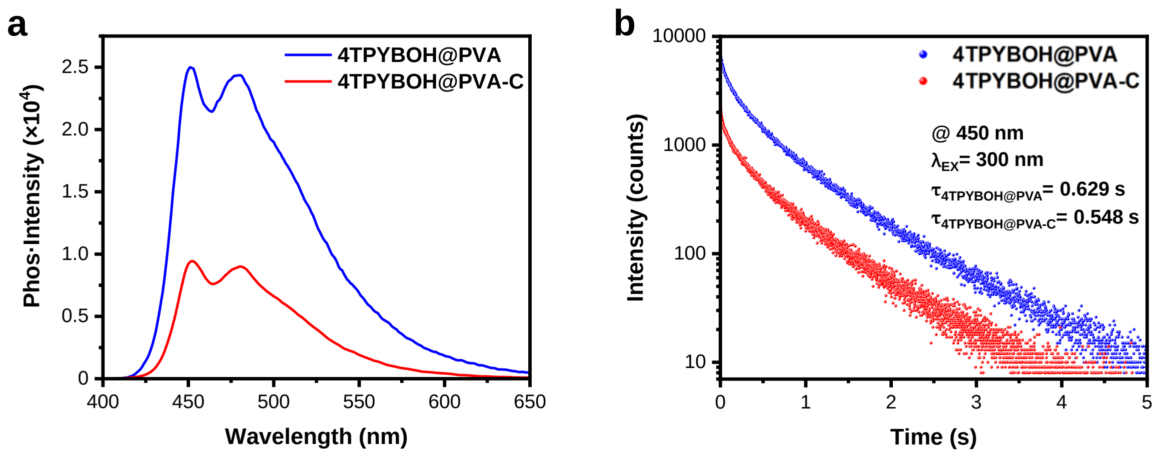

Supplementary Figure 5. (a) Phosphorescence spectra and (b) Phosphorescence-decay profiles of 0.6 wt% doped 4TPYBOH@PVA and 4TPYBOH@PVA-C films.

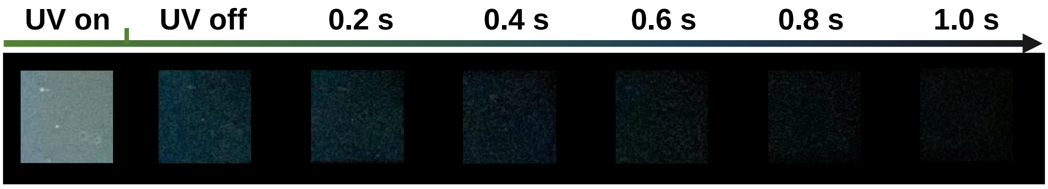

Supplementary Figure 6. Photographs of Ph-TPY@PVA film taken under 254 nm UV lamp and with the UV lamp off.

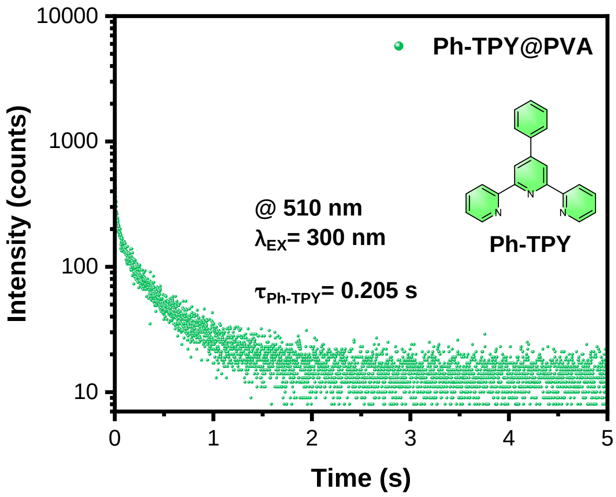

Supplementary Figure 7. Phosphorescence-decay profiles of Ph-TPY@PVA film at 510 nm.

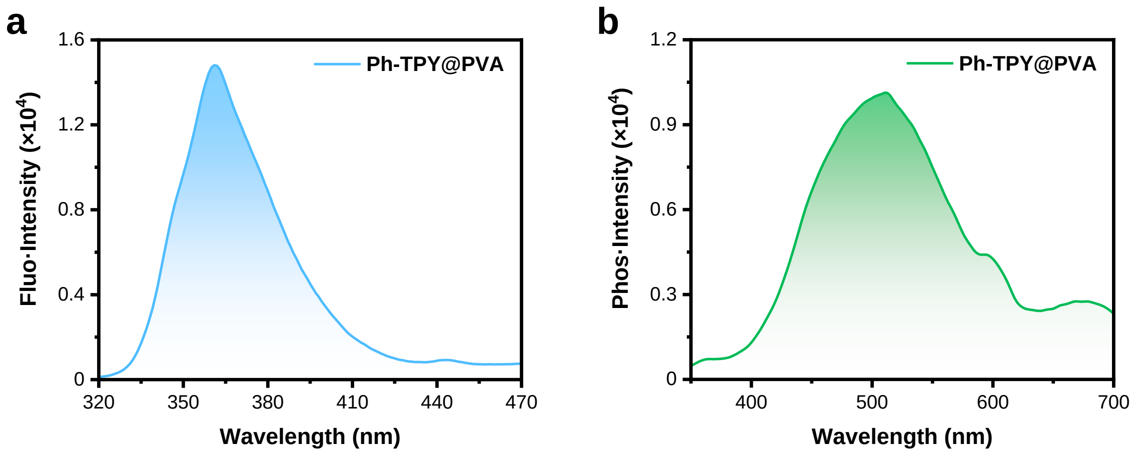

Supplementary Figure 8. (a) Fluorescence spectrum and (b) Phosphorescence spectrum of Ph-TPY@PVA film.

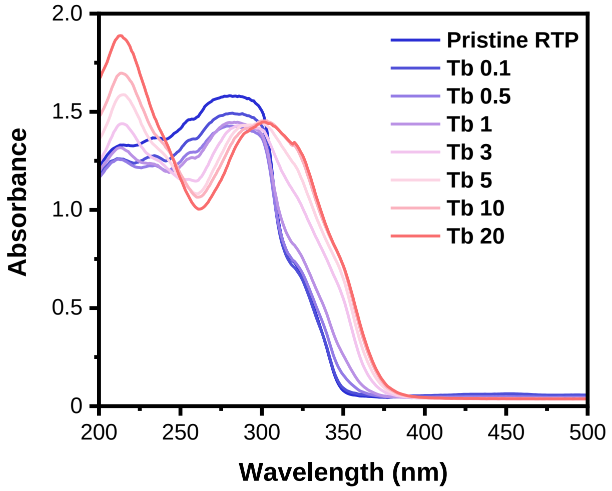

Supplementary Figure 9. UV-vis absorption spectra of 4TPYBOH@PVA-Tb films that were prepared by being immersed in different Tb^{3+} aqueous (0.1 to 20 mg/mL).

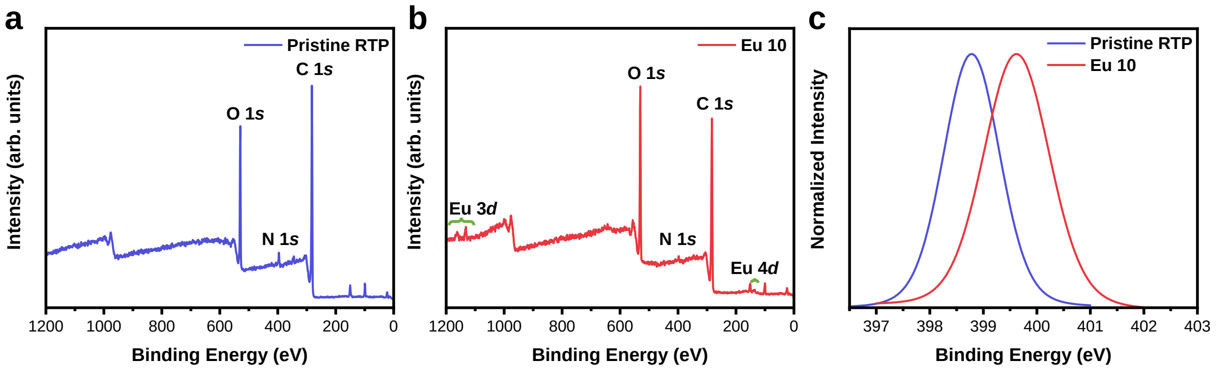

Supplementary Figure 10. XPS spectra of (a) 4TPYBOH@PVA and (b) 4TPYBOH@PVA-Eu 10. (c) High-resolution XPS spectra (N 1*s*) of 4TPYBOH@PVA and 4TPYBOH@PVA-Eu 10.

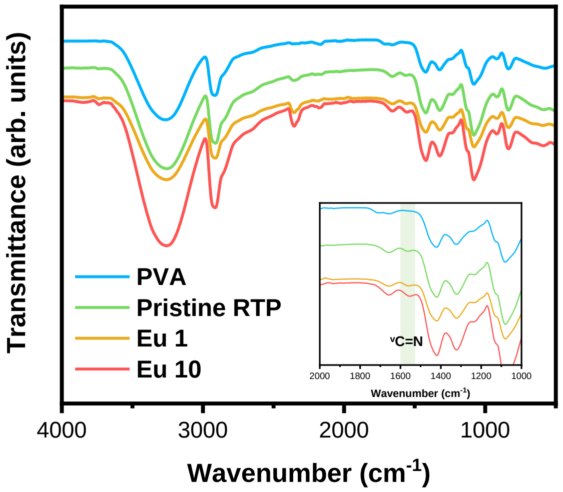

Supplementary Figure 11. ATP-FTIR spectra of PVA, 4TPYBOH@PVA, 4TPYBOH@PVA-Eu 1, and 4TPYBOH@PVA-Eu 10 RTP films.

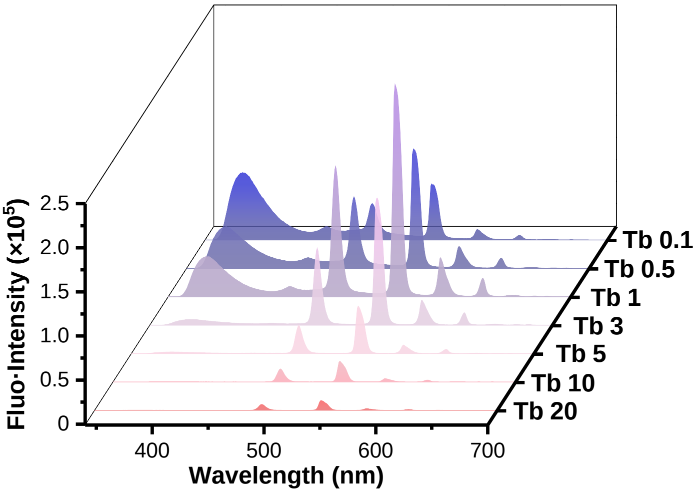

Supplementary Figure 12. Fluorescence spectra of 4TPYBOH@PVA-Tb films that were prepared by being immersed in different Tb^{3+} aqueous (0.1 to 20 mg/mL).

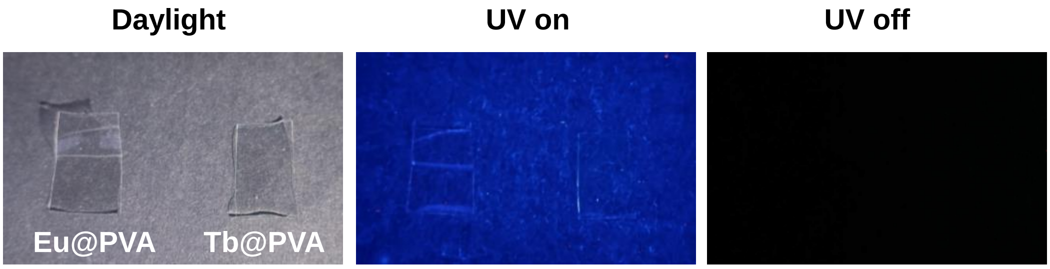

Supplementary Figure 13. Photographs of Eu@PVA and Tb@PVA films taken under daylight, 254 nm UV lamp and the removal of UV lamp.

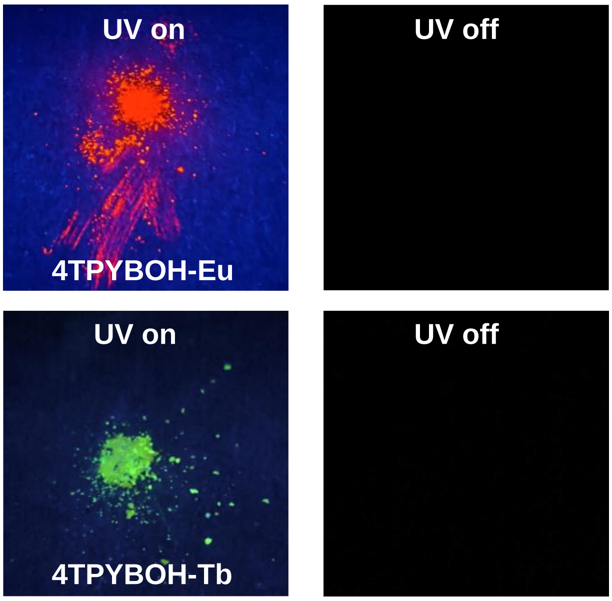

Supplementary Figure 14. Photographs of 4TPYBOH-Eu and 4TPYBOH-Tb powders taken under 254 nm UV lamp and the removal of UV lamp.

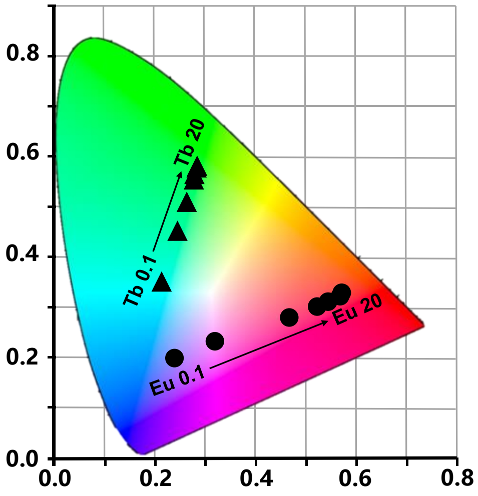

Supplementary Figure 15. CIE 1931 chromaticity diagram of corresponding the full-color luminescent coordinates of 4TPYBOH@PVA-Ln films.

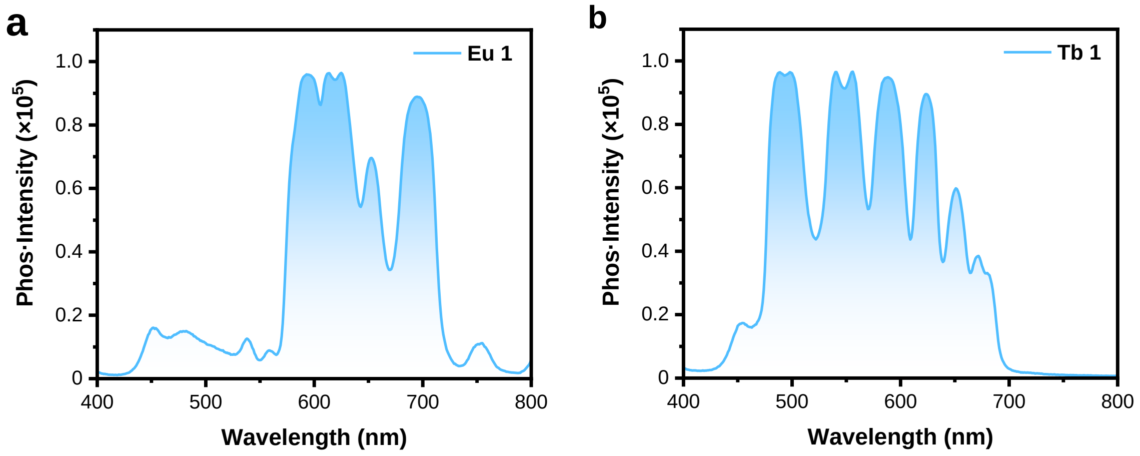

Supplementary Figure 16. Phosphorescence spectra of (a) 4TPYBOH@PVA-Eu and (b) 4TPYBOH@PVA-Tb films that were prepared by being immersed in 1 mg/mL Eu^{3+} or Tb^{3+} aqueous with a delay time of 0.05 ms.

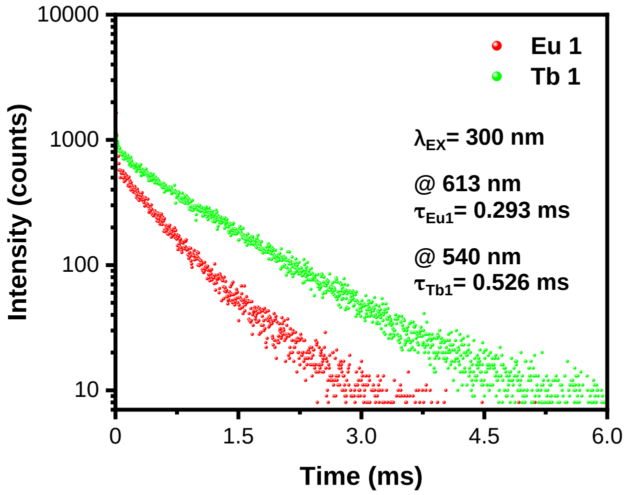

Supplementary Figure 17. Phosphorescence-decay profiles of 4TPYBOH@PVA-Eu and 4TPYBOH@PVA-Tb films that were prepared by being immersed in 1 mg/mL Eu^{3+} or Tb^{3+} aqueous.

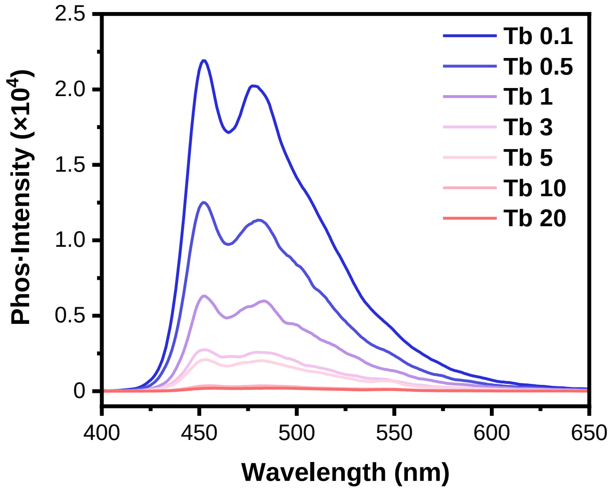

Supplementary Figure 18. Phosphorescence spectra of 4TPYBOH@PVA-Tb films that were prepared by being immersed in different Tb^{3+} aqueous (0.1 to 20 mg/mL).

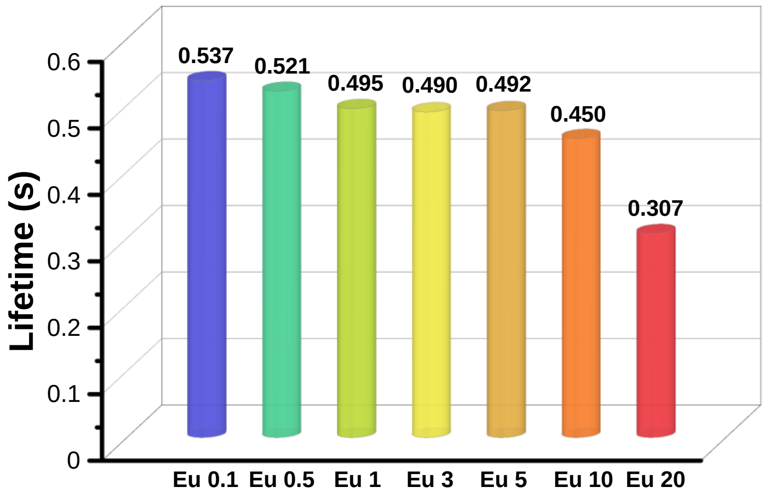

Supplementary Figure 19. Lifetime histogram (monitored at 450 nm) of 4TPYBOH@PVA-Eu films that were prepared by being immersed in different Eu^{3+} aqueous (0.1 to 20 mg/mL).

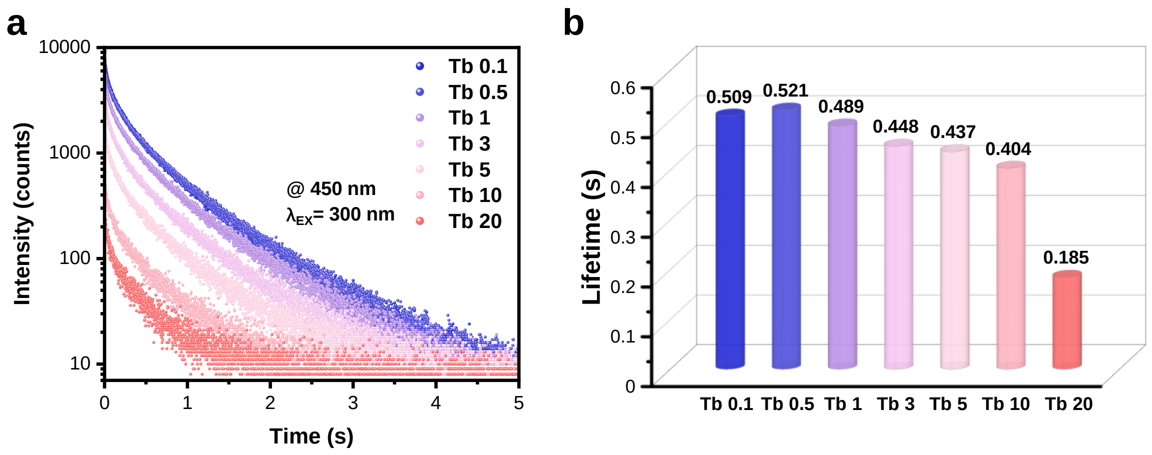

Supplementary Figure 20. (a) Phosphorescence-decay profiles and (b) Lifetime histogram (monitored at 450 nm) of 4TPYBOH@PVA-Tb films that were prepared by being immersed in different Tb^{3+} aqueous (0.1 to 20 mg/mL).

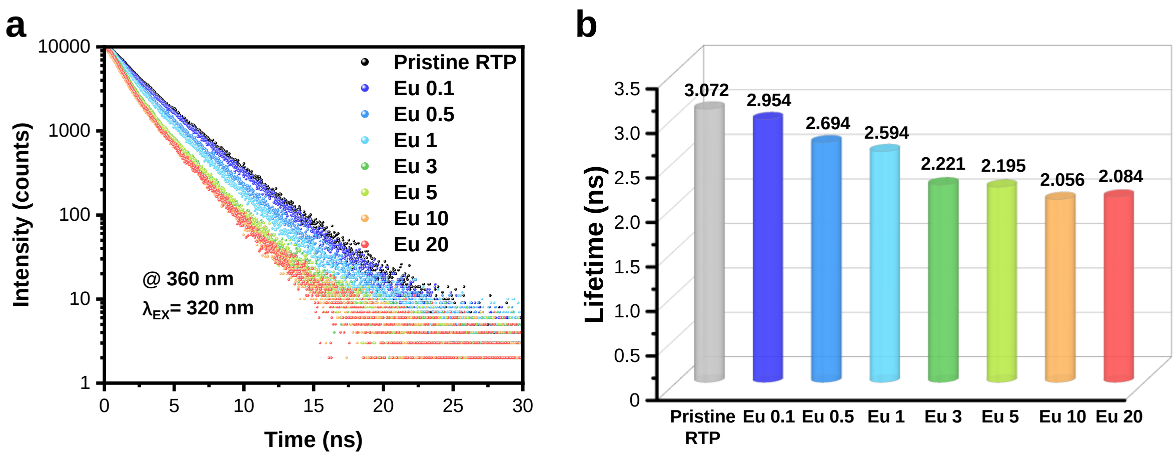

Supplementary Figure 21. (a) Fluorescence-decay profiles, and (b) Lifetime histogram (monitored at 360 nm) of 4TPYBOH@PVA-Eu films that were prepared by being immersed in different Eu^{3+} aqueous (0.1 to 20.0 mg/mL).

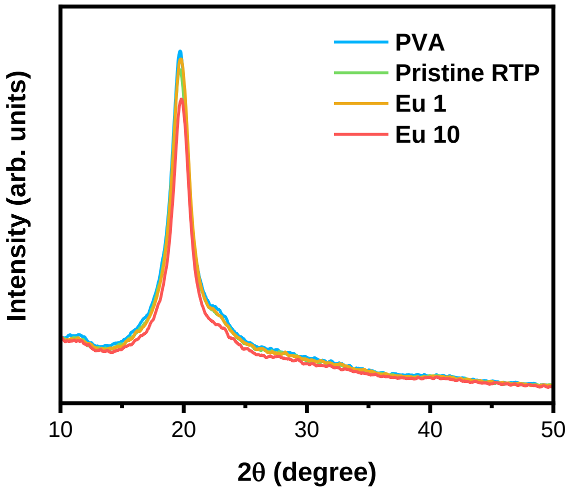

Supplementary Figure 22. Powder XRD patterns of PVA, 4TPYBOH@PVA, 4TPYBOH@PVA-Eu 1 and 4TPYBOH@PVA-Eu 10 RTP films.

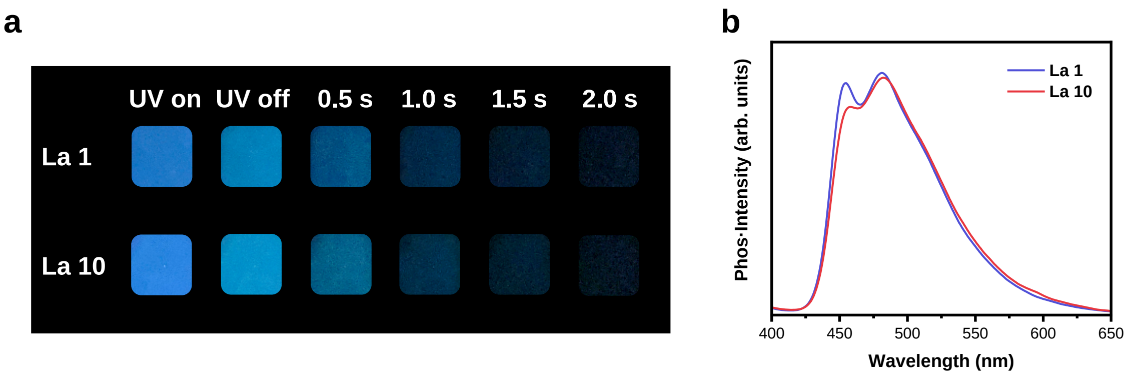

Supplementary Figure 23. (a) The photographs of La^{3+}-doped RTP films (prepared by soaking 4TPYBOH@PVA in 1.0 and 10.0 mg/mL of La^{3+} aqueous solutions, respectively), the photos were taken under 254 nm UV excitation and at different time intervals after the removal of UV irradiation. (b) Phosphorescence spectra of corresponding La^{3+}-doped RTP films.

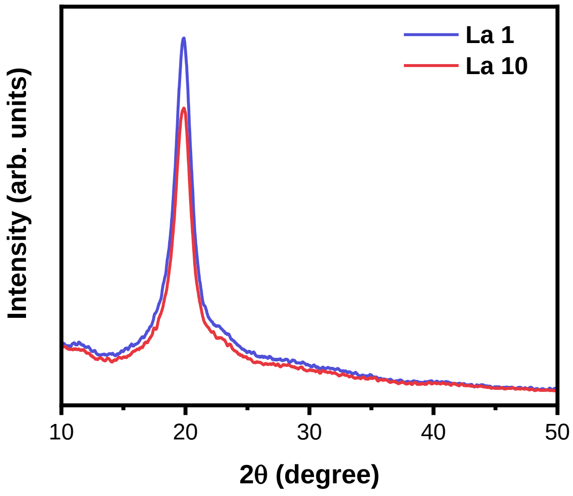

Supplementary Figure 24. Powder XRD patterns of 4TPYBOH@PVA-La 1 and 4TPYBOH@PVA-La 10.

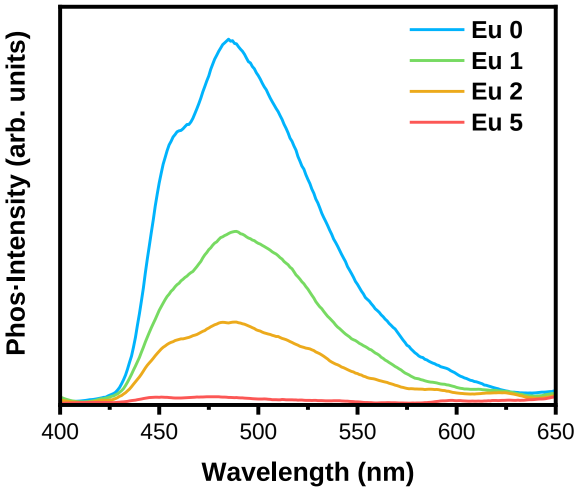

Supplementary Figure 25. Phosphorescence spectra of 4TPYBOH@PAN-Eu with different doping weights, the numbers represent 4TPYBOH@PAN doped with 0 mg, 1.0 mg, 2.0 mg and 5.0 mg of $Eu(NO_3)_3{\cdot}6H_2O$, respectively.

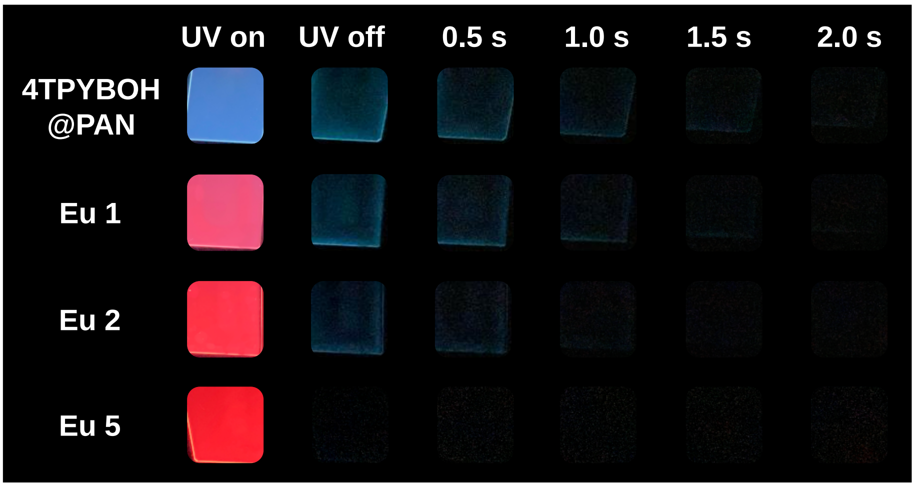

Supplementary Figure 26. The photographs of 4TPYBOH@PAN-Eu RTP films at different doping weights of $Eu(NO_3)_3 \cdot 6H_2O$ were taken under and after removing the 254 nm UV irradiation.

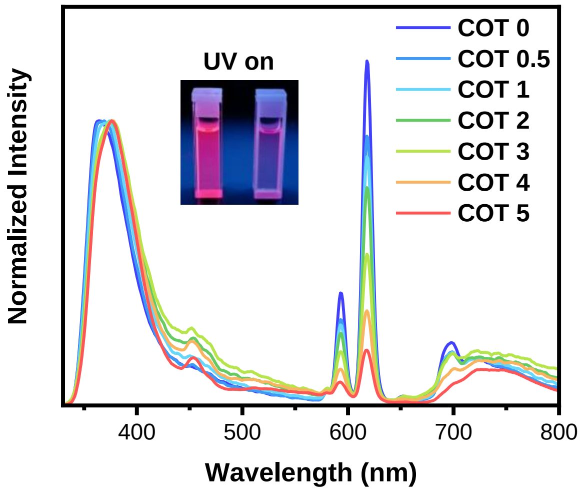

Supplementary Figure 27. Fluorescence spectra of $(4TPYBOH)_2$-Eu^{3+} without and with the addition of triplet quencher COT, λ_{ex} = 254 nm, numbers represent the volume (unit: μL) of COT additions. The Insets: the photograph of $(4TPYBOH)_2$-Eu^{3+} without and with adding 5 μL of COT under 254 nm UV light.

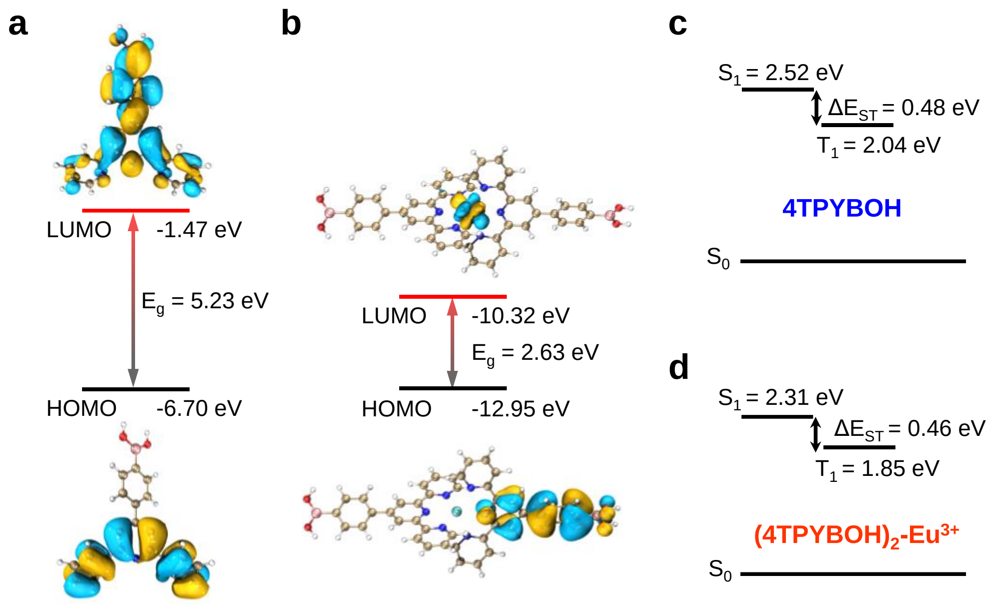

Supplementary Figure 28. The DFT calculation of the organic phosphors and lanthanide complexes. (a,b) Calculated frontier molecular orbitals and orbital energies of 4TPYBOH and $(4TPYBOH)_2-Eu^{3+}$, respectively. (c,d) Vertical excitation energy levels of 4TPYBOH and $(4TPYBOH)_2-Eu^{3+}$, respectively.

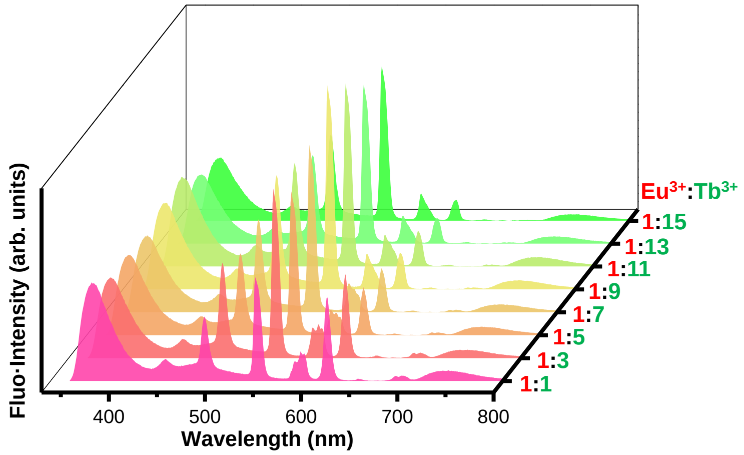

Supplementary Figure 29. Fluorescence spectra of the films simultaneously introduced Eu^{3+} and Tb^{3+}, the total concentration of immersion is fixed at 1.0 mg/mL.

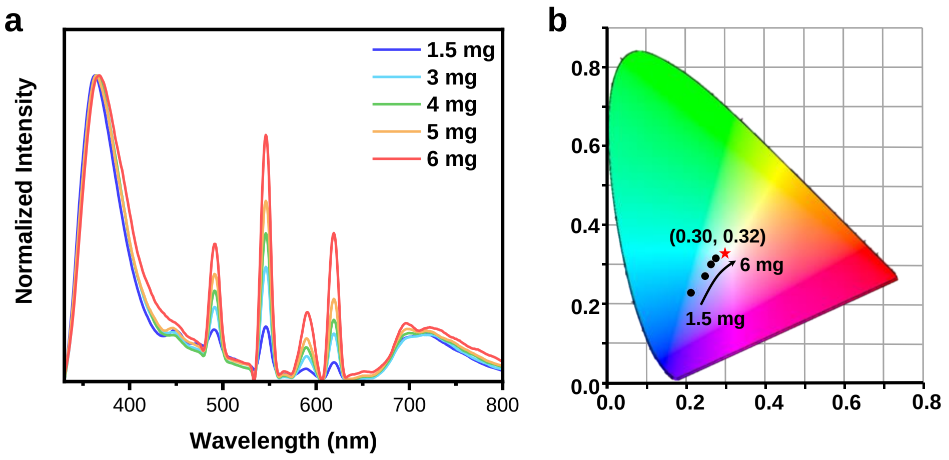

Supplementary Figure 30. (a) Fluorescence spectrum and (b) Corresponding CIE coordinate of 4TPYBOH@PVA-Ln (the molar ratio of Eu^{3+} and Tb^{3+} is 1:3 with a total mass of 1.5 mg, 3.0 mg, 4.0 mg, 5.0 mg and 6.0 mg, respectively), λ_{ex} = 254 nm.

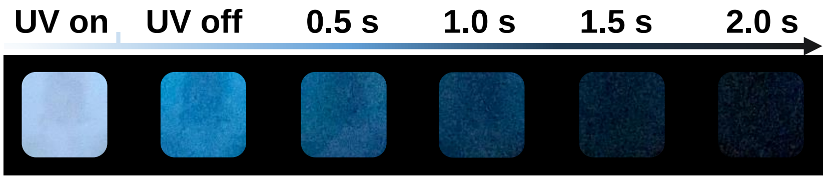

Supplementary Figure 31. The photographs of 4TPYBOH@PVA-Ln with white light emission were taken under and after removing the 254 nm UV irradiation.

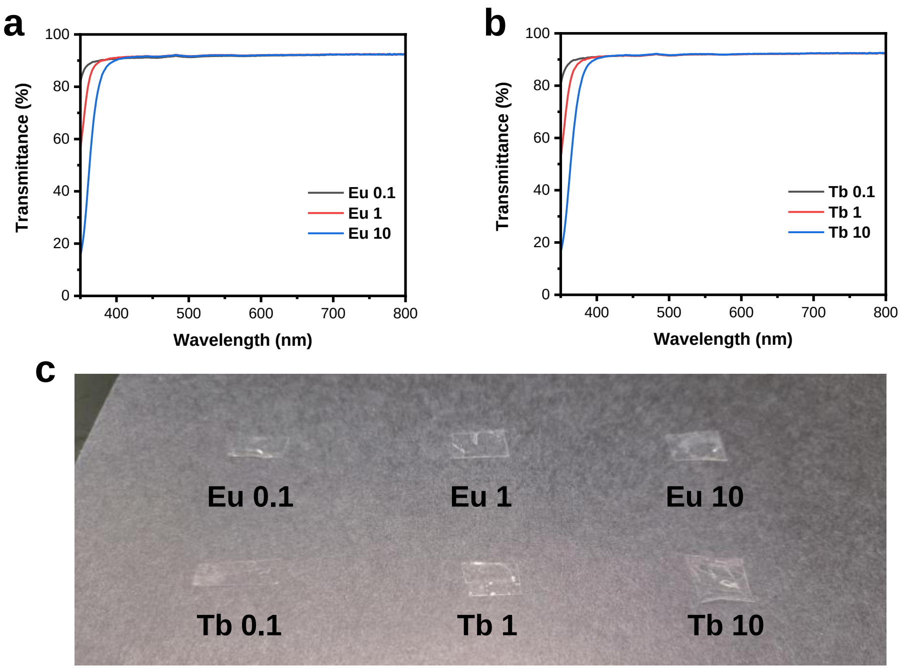

Supplementary Figure 32. Transmittance spectra of (a) 4TPYBOH@PVA-Eu and (b) 4TPYBOH@PVA-Tb films that were prepared by being immersed in different Eu^{3+} or Tb^{3+} aqueous (0.1, 1.0, 10.0 mg/mL). (c) The above films were observed under ambient conditions.

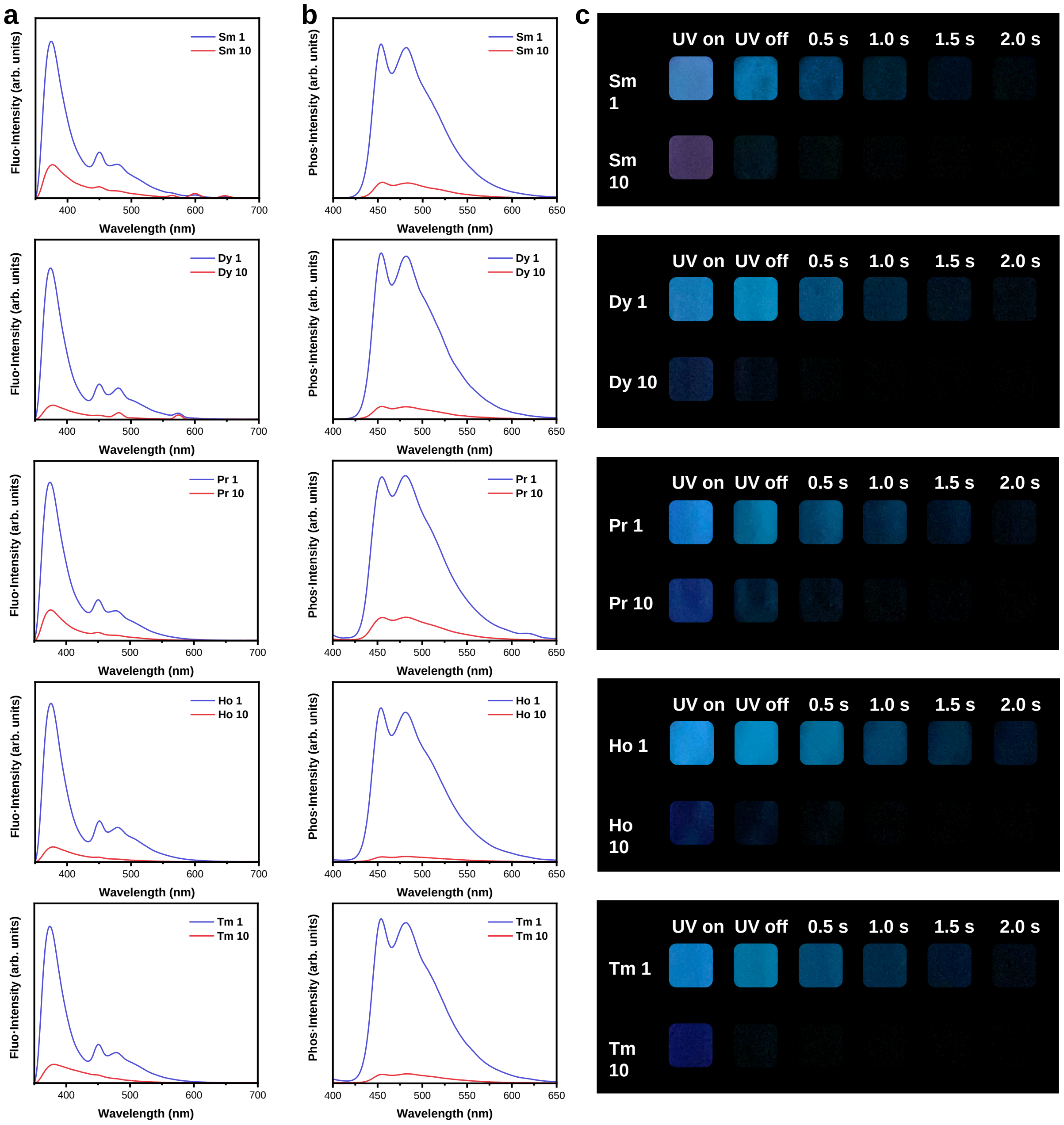

Supplementary Figure 33. (a)The fluorescence and (b) phosphorescence spectra of Sm^{3+}-, Dy^{3+}-, Pr^{3+}-, Ho^{3+}-, and Tm^{3+}-doped RTP films that were prepared by being immersed in Ln^{3+} aqueous solution (1.0 and 10.0 mg/mL). (c) The photographs of Sm^{3+}-, Dy^{3+}-, Pr^{3+}-, Ho^{3+}-, and Tm^{3+}-doped RTP films that were prepared by being soaked in Ln^{3+} aqueous solution (1.0 and 10.0 mg/mL, respectively) taken under and after removing the 254 nm UV irradiation.

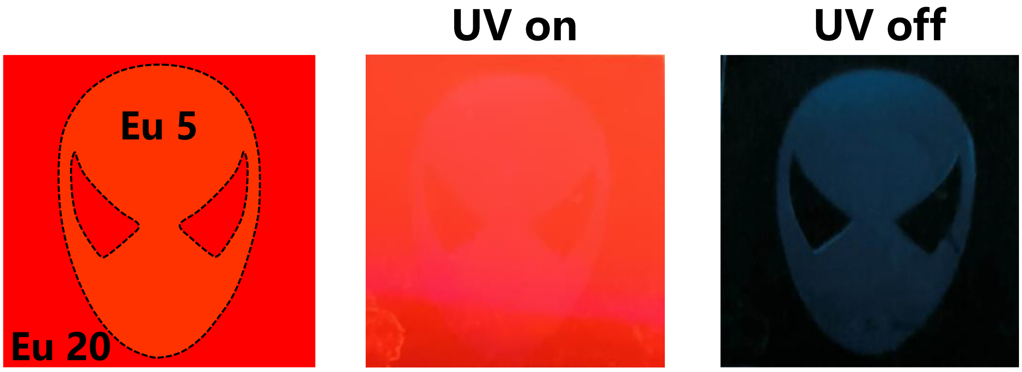

Supplementary Figure 34. The encrypted Spider-Man pattern is hidden with similar fluorescence and displayed by afterglow.

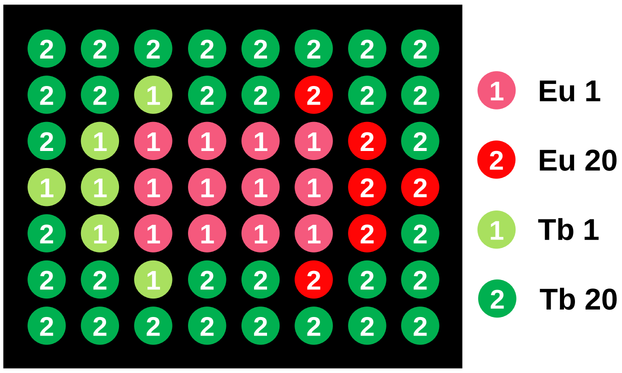

Supplementary Figure 35. The design of the disguised arrow pattern.

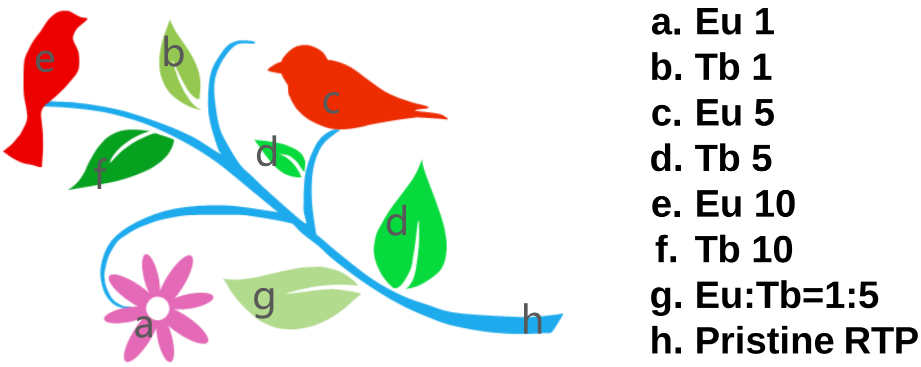

Supplementary Figure 36. The design of a flower-and-bird anti-counterfeiting pattern.

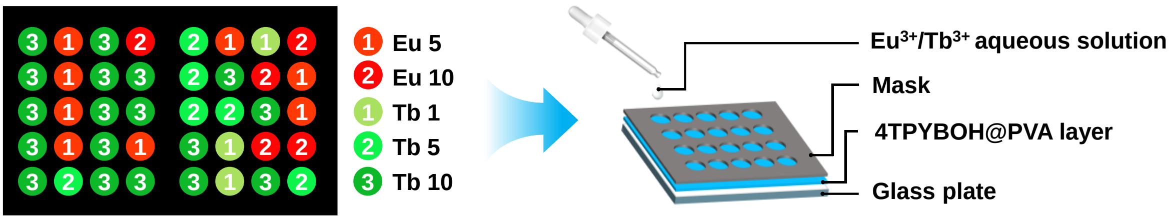

Supplementary Figure 37. The design and preparation of the encrypted dot matrix (8×5).

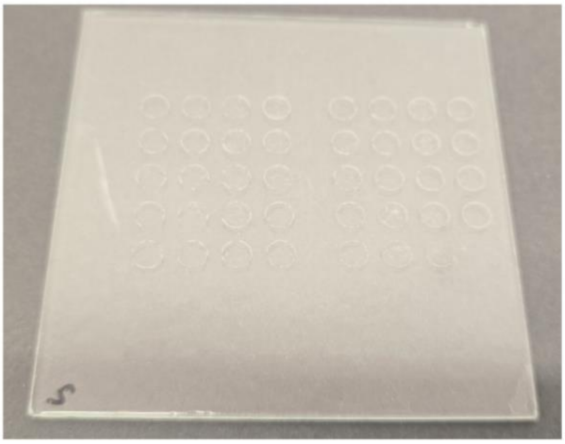

Supplementary Figure 38. The encrypted dot matrix (8×5) was observed under ambient conditions.

3. Supplementary References

1. Adamo, C. & Barone, V. Toward reliable density functional methods without adjustable parameters: The PBE0 model. *J. Chem. Phys.* **110**, 6158-6170 (1999).
2. Petersson, G. A., Tensfeldt, T. G. & Montgomery, J. A., Jr. A complete basis set model chemistry. III. The complete basis set-quadratic configuration interaction family of methods. *J. Chem. Phys.* **94**, 6091-6101 (1991).
3. Cao, X., Dolg, M. & Stoll, H. Valence basis sets for relativistic energy-consistent small-core actinide pseudopotentials. *J. Chem. Phys.* **118**, 487-496 (2003).
