# Supplementary material for: Finely manipulating room temperature phosphorescence by dynamic lanthanide coordination toward multi-level information security
Source: Nat Commun. 2024 May 8;15:3846. doi: 10.1038/s41467-024-47674-x (PMC11078970; doi:10.1038/s41467-024-47674-x)
Supplement: Supplementary file 3 — Description of Additional Supplementary Files [file 41467_2024_47674_MOESM3_ESM.pdf]

Description of Additional Supplementary Files

File Name: Supplementary Data 1
Description: The atomic coordinates of the optimized computational models.

File Name: Supplementary Movie 1
Description: Mis-leading information with arrow pattern.

File Name: Supplementary Movie 2
Description: The spatial-time-resolved anti-counterfeiting.

File Name: Supplementary Movie 3
Description: Multi-level encrypted dot matrix (8×5).
